# Investigation of the Itinerant Electron Ferromagnetism of Ni_2+*x*_MnGa_1−*x*_ and Co_2_VGa Heusler Alloys

**DOI:** 10.3390/ma12040575

**Published:** 2019-02-14

**Authors:** Takuo Sakon, Yuhi Hayashi, Akihito Fukuya, Dexin Li, Fuminori Honda, Rie Y. Umetsu, Xiao Xu, Gendo Oomi, Takeshi Kanomata, Tetsujiro Eto

**Affiliations:** 1 Department of Mechanical and Systems Engineering, Faculty of Science and Technology, Ryukoku University, Otsu, Shiga 520–2194, Japan; t150289@mail.ryukoku.ac.jp (Y.H.); t150299@mail.ryukoku.ac.jp (A.F.); 2 Institute for Materials Research, Tohoku University, Oarai, Ibaraki 311–1313, Japan; dxli@imr.tohoku.ac.jp (D.L.); honda@imr.tohoku.ac.jp (F.H.); 3 Institute for Materials Research, Tohoku University, Sendai, Miyagi 980–8577, Japan; rieume@imr.tohoku.ac.jp; 4 Department of Materials Science, Graduate School of Engineering, Tohoku University, Sendai, Miyagi 980–8579, Japan; xu@material.tohoku.ac.jp; 5 Kurume Institute of Technology, Kurume, Fukuoka 830–0052, Japan; geomi@kurume-it.ac.jp (G.O.); teto@kurume-it.ac.jp (T.E.); 6 Research Institute for Engineering and Technology, Tohoku Gakuin University, Tagajo, Miyagi 985–8537, Japan; kanomata@mail.tohoku-gakuin.ac.jp

**Keywords:** ferromagnetic Heusler alloy, magnetization, itinerant electron ferromagnetism, half-metal

## Abstract

Experimental investigations into the field dependence of magnetization and temperature dependences of magnetic susceptibility in Ni_2+*x*_MnGa_1−*x*_ (*x* = 0.00, 0.02, 0.04) and Co_2_VGa Heusler alloy ferromagnets were performed following the spin fluctuation theory of itinerant ferromagnetism, called as “Takahashi theory”. We investigated the magnetic field dependence of magnetization at the Curie temperature *T*_C_, which is the critical temperature of the ferromagnetic–paramagnetic transition, and also at *T* = 5 K, which concerns the ground state of the ferromagnetic state. The field dependence of the magnetization was analyzed by means of the *H* vs. *M*^5^ dependence, and the field dependence of the ground state at 5 K was investigated by means of an Arrott plot (*H/M* vs. *M*^2^) according to the Takahashi theory. As for Ni_2+*x*_MnGa_1−*x*_, the spin fluctuation parameter in *k*-space (momentum space, *T*_A_) and that in energy space (*T*_0_) obtained at *T*_C_ and 5 K were almost the same. On the contrary, as for Co_2_VGa, the *H* vs. *M*^5^ dependence was not shown at *T*_C_. We obtained *T*_A_ and *T*_0_ by means of an Arrott plot at 5 K. We created a generalized Rhodes–Wohlfarth plot of *p*_eff_/*p*_S_ versus *T*_C_/*T*_0_ for the other ferromagnets. The plot indicated that the relationship between *p*_eff_/*p*_S_ and *T*_0_/*T*_C_ followed Takahashi’s theory. We also discussed the spontaneous magnetic moment at the ground state, *p*_S_, which was obtained by an Arrott plot at 5 K and the high temperature magnetic moment, *p*_C_, at the paramagnetic phase. As for the localized ferromagnet, the *p*_C_/*p*_S_ was 1. As for weak ferromagnets, the *p*_C_/*p*_S_ was larger than 1. In contrast, the *p*_C_/*p*_S_ was smaller than 1 by many Heusler alloys. This is a unique property of Heusler ferromagnets. Half-metallic ferromagnets of Co_2_VGa and Co_2_MnGa were in accordance with the generalized Rhodes–Wohlfarth plot with a *k*_m_ around 1.4. The magnetic properties of the itinerant electron of these two alloys appeared in the majority bands and was confirmed by Takahashi’s theory.

## 1. Introduction

Spin fluctuation theories have been proposed to explain the physical principles of the itinerant electron system [1,2,3,4,5,6,7]. Takahashi proposed the self-consistent renormalization (SCR) theory according to zero-point spin fluctuations, which assimilated both the transverse and longitudinal components of the fluctuations [4,5,6,7]. An outstanding characteristic of this theory is the magnetization at *T*_C_. The theory proposed by Takahashi indicates that the magnetic field dependence, *H*, is proportional to the magnetization, *M*^5^, at the Curie temperature, *T*_C_. This property was obtained by the differential calculus of the magnetization of the spin fluctuation free energy [7,8,9]. 

In this theory, the relation between the magnetic fields *H* and magnetization *M* is obtained theoretically by the equation of,

(1) $$H=c(T_{C})M^{5}$$

where *c* (*T*_C_) is the constant value at *T*_C_ (refer to the references for the derivation process of Equation (1) [7,9]). MnSi [10], Fe*_x_*Co_1−*x*_Si [11], CoS_2_ [12], and Ni [13] followed the relationship provided in Equation (1). The Heusler isotropic ferromagnetic alloy Ni_2+*x*_MnGa_1−*x*_ (*x* = 0.00, 0.02, 0.04) also followed the relationship mentioned in Equation (1) [8,9,13]. From the spontaneous magnetic moment and magnetization at *T*_C_, we obtained the spin fluctuation parameter in *k*-space (momentum space, *T*_A_) and in energy space (*T*_0_).

The other approach to obtain *T*_A_ and *T*_0_ is the analysis of the field dependence of the magnetization by means of an Arrott plot (*H/M* vs. *M*^2^) at the ground magnetic state, *T* = 5 K [7,14]. Tateiwa et al. mentioned the derivation method of this approach in detail [14]. The magnetization in the ground state is expressed by the following equation

(2) $$H=\frac{F_{1}}{N_{0}^{3}{\left(g\mu_{B}\right)}^{4}}\times \left(-M_{0}^{2}+M^{2}\right)M$$

where g is Lande’s g-factor; *N*_0_ is Avogadro’s number; and *F*_1_ is the mode–mode coupling term defined as

(3) $$F_{1}=\frac{2T_{A}^{2}}{15cT_{0}}$$

where *c* is equal to 1/2 and *M*_0_ is the spontaneous magnetization. *F*_1_ is derived from the slope of the Arrott plot (*H/M* vs. *M*^2^ plot) at low temperatures by Equation (4)

(4) $$F_{1}=\frac{N_{0}^{3}{\left(2\mu_{B}\right)}^{4}}{k_{B}\zeta }$$

where *k*_B_ is the Boltzmann constant, and ζ is the slope of the Arrott plot. *T*_A_ and *T*_0_ are obtained by the following relations of

(5) $${\left(\frac{T_{C}}{T_{0}}\right)}^{5/6}=\frac{p_{S}^{2}}{5g^{2}C_{4/3}}\times {\left(\frac{15cF_{1}}{2T_{C}}\right)}^{1/2}$$

(6) $${\left(\frac{T_{C}}{T_{A}}\right)}^{5/6}=\frac{p_{S}^{2}}{5g^{2}C_{4/3}}\times {\left(\frac{2T_{C}}{15cF_{1}}\right)}^{1/2}$$

where *C*_4/3_ = 1.00608, and *p*_S_ is the spontaneous magnetic moment at the ground state (*T* = 0 K). In the Takahashi theory, it is mentioned that the experimental results of the magnetization measurement can be applied to these equations in units of kOe and emu/g for the magnetic fields *H* and magnetization *M*, respectively (p. 66 in Reference [7]). Therefore, we used these units to calculate the *T*_A_ and *T*_0_ parameters clearly. Incidentally, the value of the magnetic field *H* in 10 kOe is equal to the value in T (Tesla), and the value of magnetization *M* in emu/g is equal to the value in Am^2^/kg.

Tateiwa et al. evaluated the parameters, *T*_A_ and *T*_0_, of actinide 5f electron systems which were analyzed by means of Equations (4)–(6) [14]. Tateiwa et al. also used the units of kOe and emu/g.

The relation between *p*_S_, *T*_C_, *T*_0_, and the effective magnetic moment *p*_eff_ in the paramagnetic phase was derived from a formula shown in Equation (3.47) in [7], as follows:

(7) $$\frac{p_{\mathrm{eff}}}{p_{S}}\approx 1.4\times {\left(\frac{T_{0}}{T_{C}}\right)}^{\frac{2}{3}}$$

Equation (7) can be rewritten as:

(8) $$k_{m}=\left(\frac{p_{\mathrm{eff}}}{p_{S}}\right)\times {\left(\frac{T_{C}}{T_{0}}\right)}^{\frac{2}{3}}$$

When *k*_m_ is 1.4, Equation (8) is equal to Equation (7).

In this study, experimental investigations into the field dependence of magnetization and temperature dependences of magnetic susceptibility in Ni_2+*x*_MnGa_1−*x*_ (*x* = 0.00, 0.02, 0.04) and half-metallic ferromagnets (HMFs) of Co_2_VGa and Co_2_MnGa Heusler alloys were performed following the self-consistent renormalization (SCR) spin fluctuation theory of itinerant electron ferromagnetism by Y. Takahashi [7]. We investigated the magnetic field dependence of magnetization at the Curie temperature *T*_C_, which is the critical temperature of the ferromagnetic–paramagnetic transition, and also at *T* = 5 K, which concerns the ground state of the ferromagnetic phase. We created a generalized Rhodes–Wohlfarth plot of *p*_eff_/*p*_S_ versus *T*_C_/*T*_0_ for the other ferromagnets. The plot indicated that the relationship between *p*_eff_/*p*_S_ and *T*_C_/*T*_0_ followed Takahashi’s theory. We also discussed the magnetism of Heusler alloys by comparing the spontaneous magnetic moment *p*_S_ at the ground state (*T* = 0 K) and paramagnetic magnetic moment *p*_C_.

## 2. Materials and Methods

The polycrystalline samples of Ni_2+*x*_MnGa_1−*x*_ (*x* = 0.00, 0.02, 0.04) were prepared by arc melting the constituent elements, nominally, 4N Ni, 3N Mn, and 6N Ga, several times in an Ar atmosphere. Each ingot was melted several times to ensure good homogeneity. The products from the arc melting process were sealed in an evacuated silica tube and solution heat-treatment was applied at 1123 K for 3 days. After these treatments, the sample was quenched in water. The polycrystalline sample of Co_2_VGa was fabricated by levitation melting after making a 66.6Co–33.4Ga (at.%) binary alloy by induction furnace melting in order to avoid the reaction of the crucible by the V element. The purity of the starting elements were 99.7% V, 3N Co, and 4N Ga. The obtained ingot was annealed at 1373 K for 3 days and quenched in water.

The magnetization measurements were performed up to 5 T by means of a SQUID magnetometer (Quantum Design Inc., San Diego, USA) at the Institute for Materials Research, Tohoku University. The permeability measurement was performed in AC magnetic fields with a frequency of 73 Hz and maximum field of ± 10 Oe. The AC magnetic fields were measured by a gaussmeter 410 (Lakeshore Cryotronix Inc., Westerville, Ohio, USA). The magnetic susceptibility measurements were performed by means of a vibrating sample magnetometer (VSM, PASCO Co. Ltd, Roseville, CA, USA), which was installed in a water-cooled electromagnet (Tamagawa Seisakusho Co. Ltd., Sendai, Japan) at Ryukoku University. The magnetic susceptibility χ in the paramagnetic phase was obtained from the temperature dependences of magnetization *M*, measured at the magnetic fields of *H* = 0.10 T and the relation of χ=M/H. 

## 3. Results and Discussion

### 3.1. Results of the Magnetic Measurements of Ni_2+x_MnGa_1−x_

**Figure 1:**
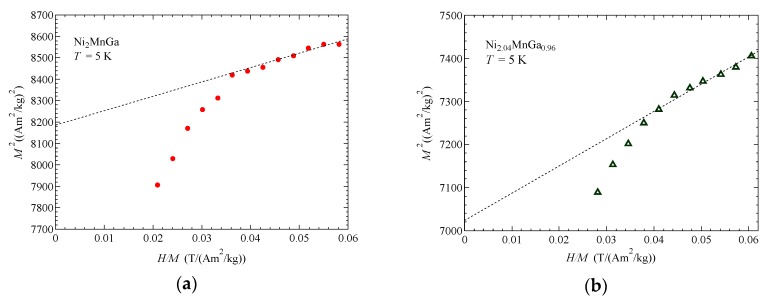
Arrott plot (*M*^2^ vs. *H/M*) of: (**a**) Ni_2_MnGa and (**b**) Ni_2.04_MnGa_0.96_ at *T* = 5 K.

Figure 1 shows the Arrott plot (*M*^2^ vs. *H/M*) of: (a) Ni_2_MnGa, and (b) Ni_2.04_MnGa_0.96_ at *T* = 5 K. By using the slope value ζ of the Arrott plot, the parameter *F*_1_ was derived by Equation (4). The spontaneous magnetic moment, *p*_S_; effective moment, *p*_eff_; Curie temperature, *T*_C_; and spin fluctuation parameters *T*_A_ and *T*_0_ are listed in Table 1. The obtained *T*_A_ and *T*_0_ by the relations of Equations (5) and (6) are also listed in Table 1. Errors of *T*_A_ and *T*_0_ were estimated as ±10%, which arose from the error of fitting of the Arrott plot. Within these errors, the *T*_A_ and *T*_0_ obtained from a low temperature and the values from *T*_C_ were the same as each other.

In a previous study, we analyzed the results of Ni_2_MnGa by means of the generalized Rhodes–Wohlfarth plot (double logarithmic plot of *p*_eff_/*p*_S_ and *T*_C_/*T*_0_) [9], which was derived to formulate the magnetic moments ratio, *p*_eff_/*p*_S_, and the critical temperature ratio, *T*_C_/*T*_0_. Takahashi derived an equation for the relationship between *p*_S_, *T*_C_, *T*_0_ and the effective magnetic moment *p*_eff_ as Equation (7). As for Ni_2_MnGa, the measured effective moment *p*_eff_, which was measured in this work, was 4.75, which was the same value as the result by Webster et al. [15]. For Ni_2_MnGa, a value of 1.61 for *k*_m_ was obtained by substituting a *p*_eff_ of 4.75, and *p*_S_, *T*_C_, and *T*_0_ from Table 1 into Equation (8).

In order to investigate the *k*_m_ values of Ni_2+*x*_MnGa_1−*x*_ (*x* = 0.02, 0.04) and compare them with other ferromagnetic alloy and compounds, we further needed *p*_eff_ values of these alloys. We measured the magnetic susceptibility of these alloys, and *p*_eff_ values were obtained from the Curie constant of the Curie law. 

**Figure 2:**
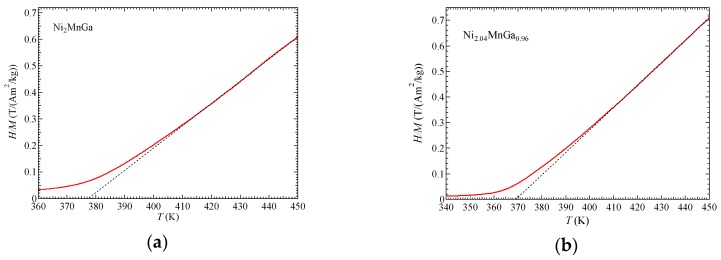
Inverse magnetic susceptibilities as: (**a**) Ni_2_MnGa and (**b**) Ni_2.04_MnGa_0.96_. Dotted lines are the fitting lines at the paramagnetic phase.

Figure 2 shows the inverse magnetic susceptibilities, 1/χ=H/M. The gradient of 1/χ vs. *T*, which is indicated by the dotted lines, is equal to 1/*C*, where *C* is a Curie constant.

The Curie constant *C* is written as:

(9) $$C=\frac{Np_{\mathrm{eff}}^{2}\mu_{B}^{2}}{3k_{B}}$$

where *N* is the molecular number per gram. The obtained effective moments *p*_eff_ were 4.72 for Ni_2.02_MnGa_0.98_ and 4.68 for Ni_2.04_MnGa_0.96_. In Section 3.3, we discuss the itinerant electron ferromagnetism by means of these parameters.

### 3.2. Results of the Magnetic Measurements of Half-Metallic Ferromagnet Co_2_VGa

HMFs are comprised of a metallic band for one spin direction. For the other spin direction, a semiconducting band has an energy gap around Fermi energy. Co_2_VGa is a HMF with a high spin polarization [16]. It has an *L*2_1_-type cubic crystal structure with a lattice constant *a* = 0.5782 nm. The spin polarization ratio *P* is defined as:

(10) $$P_{0}\left(\%\right)=\left|\frac{N_{↑}\left(E_{F}\right)-N_{↓}\left(E_{F}\right)}{N_{↑}\left(E_{F}\right)+N_{↓}\left(E_{F}\right)}\right|\times 100$$

where $N_{↑}\left(E_{F}\right)$ and $N_{↓}\left(E_{F}\right)$ denote the density of states, DOS, at the Fermi energy, *E_F_*, in the majority spin (↑) and minority spin (↓), respectively. Umetsu et al. calculated the DOS by means of the LTMO method with the atomic spheres approximation (ASA). From the results of this calculation, the *P*_0_ value (*P* value at *T* = 0 K) was 75% and the *P*_0_ value of *L2*_1_-type Co_2_(V_1−*x*_Mn*_x_*)Ga alloys (0≤x≤1) was also determined. As for *x* = 1, Co_2_MnGa, the obtained spin polarization ratio *P*_0_ was 48%. This indicates that Co_2_VGa is a higher polarized HMF. The Curie temperatures of Co_2_VGa and Co_2_MnGa were 337 K and 695 K, respectively. We measured the magnetic field dependences of the magnetization to obtain the magnetic moments, *p*_s_, and the spin fluctuation parameters, *T*_A_ and *T*_0_, and also measured the magnetic susceptibility to obtain the effective magnetic moment, *p*_eff_, in the paramagnetic phase. We also obtained *T*_A_ and *T*_0_ of Co_2_MnGa according to the Takahashi theory by means of the magnetization process at 5 K in Reference [16].

**Figure 3:**
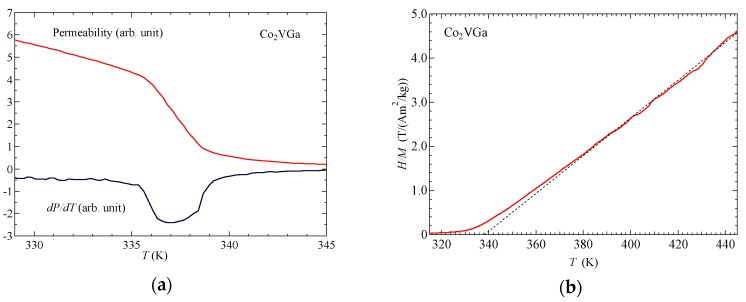
(**a**) Permeability of Co_2_VGa around the Curie temperature. *dP/dT* indicates the differential of the permeability in the temperature. (**b**) Inverse magnetic susceptibility *1/χ = H/M* of Co_2_VGa. Dotted line is a fitting line at the paramagnetic phase.

Figure 3a shows the permeability of Co_2_VGa around the Curie temperature. From the differentiation of the permeability for the temperature, denoted as *dP/dT*, the Curie temperature was obtained as *T*_C_ = 337 K. Figure 3b shows the inverse magnetic susceptibility 1/χ=H/M of Co_2_VGa. The obtained *p*_eff_ was 2.06. 

**Figure 4:**
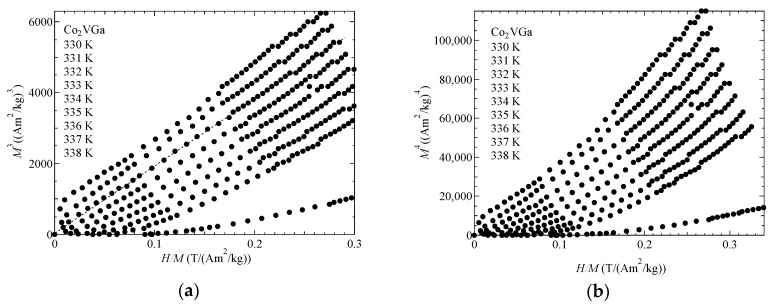
The magnetic field dependences of the magnetization of Co_2_VGa: (**a**) *M*^3^ vs. *H/M*; (**b**) *M*^4^ vs. *H/M*. Dotted straight line in (a) is a guide for the eyes.

Figure 4 shows the *M*^3^ vs. *H/M* plot and *M*^4^ vs. *H/M* plot of Co_2_VGa around *T*_C_ = 337 K.

**Figure 5:**
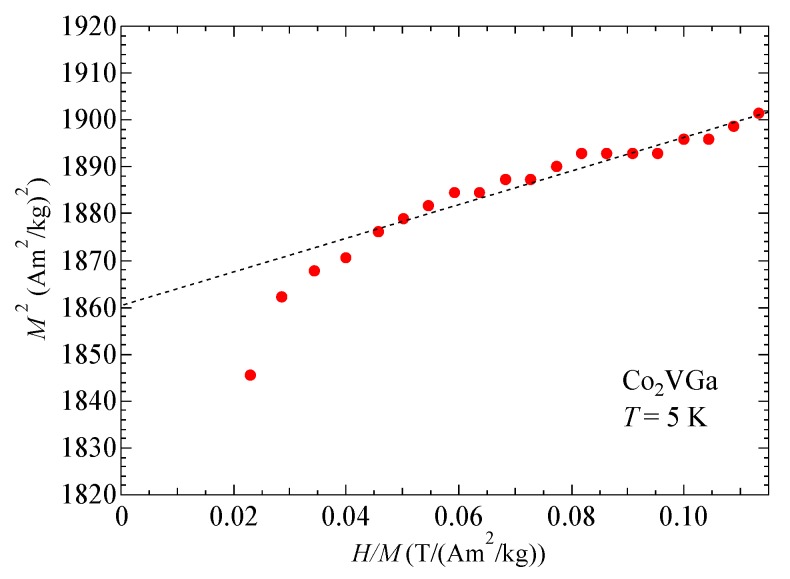
The Arrott plot (*M*^2^ vs. *H/M*) of Co_2_VGa at *T* = 5 K.

With regard to the Takahashi theory, the magnetization process is expressed as *(H/M*)∝*M*^4^ by Equation (1) around *T*_C_. On the contrary, the *H/M* was almost proportional to *M*^3^ as shown in Figure 4a. Nishihara et al. also measured the magnetization around *T*_C_ [17]. The magnetization process at *T*_C_ is expressed as *H*∝*M^D^* with the index *D* = 4.15 ± 0.05. Their result was the same as in this study. Nishihara et al. mentioned that the discrepancy between these experimental magnetization results and the Takahashi theory is supposed to arise from the distribution of *T*_C_ in the sample because the fourth-order expansion of the magnetic-free energy vanishes at the Curie temperature. In this study, we tried again with other ingots from the former sample used by Nishihara et al. As this experiment reproduced the former experiment, there may be an essential reason. Incidentally, other magnetic models have indicated that the molecular field theory denotes the *D* value as 3.0, the three-dimensional Heisenberg model denotes the *D* value as 4.8, and the three dimensional Ising model as 4.82 [18]. None of these matched the analysis in this investigation. In order to obtain the spin fluctuation parameters *T*_A_ and *T*_0_ of Co_2_VGa, we measured the magnetization process of Co_2_VGa at 5 K. Figure 5 shows the Arrott plot (*M*^2^ vs. *H/M*) of Co_2_VGa. The parameter *F*_1_ was obtained by applying the slope value of the Arrott plot to Equation (4). The parameters *T*_A_ and *T*_0_ were derived by Equations (5) and (6). The obtained *T*_A_ and *T*_0_ were 2258 K and 213 K, respectively.

### 3.3. Analysis According to the Takahashi Theory

Table 2 indicates the Curie temperature *T*_C_, the effective magnetic moment *p*_eff_, the spontaneous magnetization *p*_S_, the magnetic moment ratio *p*_eff_/*p*_S_, the spin fluctuation parameters *T*_A_ and *T*_0_, the critical temperature ratio *T*_C_/*T*_0_, and *k*_m_, as obtained from Equation (8).

**Figure 6:**
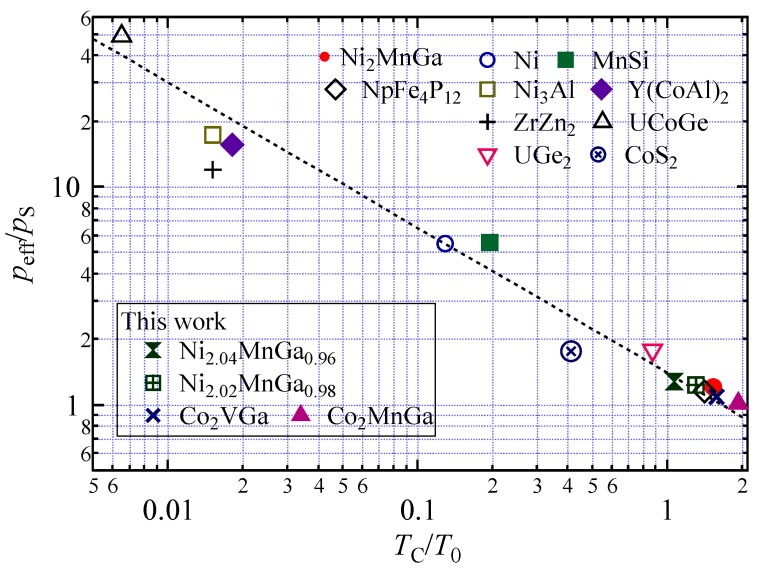
The generalized Rhodes–Wohlfarth plot (double logarithmic plot of *p*_eff_/*p*_S_ and *T*_C_/*T*_0_) for this work and other notable alloys and compounds. The dotted line indicates *k*_m_ = 1.4 as obtained from Equation (8).

The *k*_m_ value was around 1.4. Figure 6 shows the generalized Rhodes–Wohlfarth plot using the parameters in Table 2 [7,26]. The points of Ni_2+*x*_MnGa_1−*x*_ are in accordance with the dotted line as *k*_m_ = 1.4. It is noteworthy that the HMFs, Co_2_VGa and Co_2_MnGa, were also in accordance with this line. Originally, the Takahashi theory was applied to weak ferromagnets. It is interesting that this theory can be applied to strongly correlated 5*f* electron systems as well as Heusler HMFs.

### 3.4. Comparison between the Spontaneous Magnetic Moment at the Ground State, p_S_, and the Paramagnetic Magnetic Moment, p_C_, for HMFs

In this subsection, we consider the magnetism of Heusler alloys by comparing the spontaneous magnetic moment at the ground state and paramagnetic magnetic moment. 

We rewrote the definitions of *p*_S_, *p*_sat_, *p*_eff_, and *p*_C_ to make the following argument plain. *p*_S_ is the spontaneous magnetic moment at the ground state (*T* = 0 K or *T* << *T*_C_). *p*_sat_ is the saturation magnetic moment at the ground state (*T* = 0 K or *T*<< *T*_C_). *p*_eff_ is the effective magnetic moment in the paramagnetic phase. *p*_C_ is the magnetic moment in the paramagnetic phase. These four magnetic moments are defined by the unit of $\mu_{B}.$ The relation between *p*_eff_ and *p*_C_ is described as

(11) $$p_{\mathrm{eff}}=\sqrt{p_{C}\left(p_{C}+2\right)}$$

In HMFs, the band for minority spin electrons has a gap at the Fermi level and indicates semi-metallic bands. On the other hand, for majority spin electrons, the Fermi level intersects the bands and represents metallic bands. Table 3 represents the magnetic parameters of ferromagnetic Heusler alloys, with the paramagnetic moment *p*_C_. The notable point of Table 3 is that the *p*_C_/*p*_s_ of Ni_2_MnGa and many half-metallic Heusler alloys were smaller than 1. From Equation (9) and (11), the *p*_C_ is calculated by the Curie constant, *C* = *Nμ*_eff_^2^/3*k*_B_ = *Np*_eff_^2^*μ*_B_^2^/3*k*_B_ = *Np*_C_(*p*_C_ + 2)*μ*_B_^2^/3*k*_B_. *p*_C_ refers to the magnetic moment in the paramagnetic phase deduced from the Curie constant C. *p*_c_/*p*_s_ is 1 for the local moment ferromagnetism. For the weak itinerant electron ferromagnetism, the *p*_c_/*p*_s_ is larger than 1 [7]. 

As for Ni_2_MnGa, *p*_eff_ was 4.75, as shown in Table 3. Therefore, the *p*_c_ obtained was 3.85 from Equation (11), and the *p*_C_/*p*_S_ value was 0.980. As a result, the *p*_C_/*p*_S_ was a little smaller than 1. Webster et al. compared the magnetic moment obtained by the saturation magnetization measurement where *p*_sat_ = 4.17 [15]. Then, the *p*_sat_/*p*_s_ was 0.92. The magnetization of Ni_2_MnGa in the magnetic field of 5.0 T at 5 K was 4.10 *μ*_B_/f.u. Therefore, the *p*_sat_/*p*_s_ was 0.96. Regarding the half-metallic Heusler alloys, Co_2_VGa and Co_2_MnGa, which are the focus of this article, the *p*_sat_/*p*_s_ were 0.70 and 0.80, respectively. The renowned half-metallic Heusler alloys and compounds listed in Table 3 indicate the property of *p*_C_/*p*_S_ < 1. The magnetic properties of the inter-metallic compounds CoMnSb, NiMnSb, PtMnSb, Pd_2_MnSn, and Pd_2_MnSb showed an effective paramagnetic moment above *T*_C_, which was also smaller than the spontaneous and saturation moment of the ground state at *T* = 0 K [27,28]. 

As above-mentioned, the spin polarization values *P*_0_ of Co_2_VGa and Co_2_MnGa were 75% and 48%, respectively [16]. This indicates that Co_2_VGa is a higher polarized HMF. The *p*_C_/*p*_S_ values of Co_2_VGa and Co_2_MnGa were 0.70 and 0.80, respectively, as shown in Table 3. The results concerned with these two alloys indicate that the alloy with a larger spin polarization showed a smaller *p*_C_/*p*_S_ value. 

Dong et al. studied the spin polarization of Co_2_MnGe experimentally and analyzed the temperature dependence of the spin polarization [29] where the spin polarization of Co_2_MnGe was 27% at 2 K. However, the spin polarization decreased with increasing temperature and vanished at 300 K. It is considered that the magnetic moment decreases at a high temperature with the decrease of the spin polarization. Ott et al. also suggested that this effect could be attributed to a decrease of the conduction electron spin polarization in the paramagnetic phase, which has a higher temperature than *T*_C_ [27]. A simple molecular field model, which took into account both local moments and spin-polarized itinerant electrons, explained that *p*_C_/*p*_S_ < 1 [27]. They introduced an “Enhanced Temperature-independent Pauli susceptibility”, which comes from the itinerant electron bands intersecting the Fermi level, and explained that the Curie constant was reduced if the interactions between local magnetic moments and holes were antiferromagnetic. Therefore, the reduction in the Curie constant indicates that the magnetic moment *p*_C_ at a high temperature in a paramagnetic phase is smaller than that of the spontaneous magnetization *p*_S_ as well as the saturation moment *p*_sat_ at the ground phase of *T* = 5 K. Webster et al. pointed out the electronic and spin phases of Ni_2_MnGa [15]. In the paramagnetic phase, only the Mn atoms carry a magnetic moment. It is supposed that in the paramagnetic phase a large moment was induced by the electrons around the Mn atom at the Mn site. On the contrary, at the Ni site, the spins fluctuated at high temperature in the paramagnetic phase. Therefore, it is also supposed that *p*_C_ at a high temperature in the paramagnetic phase is smaller than that of the *p*_S_. As for Co_2_VGa and Co_2_MnGa, which were treated in this article, it is of interest to investigate the temperature dependence of the spin polarization experimentally.

In Figure 6, the HMFs of Co_2_VGa and Co_2_MnGa were in accordance with the generalized Rhodes–Wohlfarth plot with a *k*_m_ around 1.4. The majority of the bands of these two alloys intersected the Fermi level [16]. Therefore, the magnetic property of the itinerant electron appeared in the majority bands and was confirmed by Takahashi’s theory.

## 4. Conclusions

In this article, experimental investigations and discussions into the field dependence of magnetization and temperature dependences of magnetic susceptibility in Ni_2+*x*_MnGa_1__−*x*_ (*x* = 0.00, 0.02, 0.04), Co_2_VGa, and Co_2_MnGa Heusler alloy ferromagnets were performed following the spin fluctuation theory of itinerant electron ferromagnetism by Y. Takahashi.

1. As for Ni_2+*x*_MnGa_1__−*x*_, the spin fluctuation parameters in *k*-space (momentum space, *T*_A_) and that in energy space (*T*_0_) obtained at *T*_C_ and 5 K were almost the same within ±10% error. This consequently indicates that the spin fluctuation parameters can be obtained from the *H/M* vs. *M*^4^ plot at *T*_C_ and also from an Arrott plot (*H/M* vs. *M*^2^) at a low temperature of *T* << *T*_C_.
2. As for Co_2_VGa, the *H* vs. *M*^5^ dependence was not shown at *T*_C_. *T*_A_ and *T*_0_ were obtained by means of an Arrott plot at 5 K, which is well below *T*_C_ = 337 K;
3. In order to obtain a *k*_m_ value as defined in Equation 8, the magnetic susceptibility was measured, and the *p*_eff_ was obtained by means of Curie law. The *k*_m_ of Co_2_VGa (1.50) and Co_2_MnGa (1.57) were around 1.4, which was proposed in Takahashi’s theory. The generalized Rhodes–Wohlfarth plot of *p*_eff_/*p*_S_ versus *T*_C_/*T*_0_ indicated that the relationship between *p*_eff_/*p*_S_ and *T*_0_/*T*_C_ for the ferromagnets, including Ni_2+*x*_MnGa_1__−*x*_ and HMFs of Co_2_VGa and Co_2_MnGa, followed Takahashi’s theory. In HMFs, the band for minority spin electrons has a gap at the Fermi level and indicates semi-metallic bands. On the other hand, for majority spin electrons, the Fermi level intersects the bands and represents metallic bands. The magnetic properties of the itinerant electron of these two HMFs alloys appeared in the majority bands and were confirmed by Takahashi’s theory;
4. As for Ni_2+*x*_MnGa_1__−*x*_ and HFMs, we obtained the spontaneous magnetic moment at the ground state, *p*_S_, by an Arrott plot at 5 K, and the high temperature magnetic moment, *p*_C_, at the paramagnetic phase. The *p*_C_/*p*_S_ was smaller than 1 for many Heusler alloys, which is a different property from the localized ferromagnets (*p*_C_/*p*_S_ = 1), or, for weak itinerant electron ferromagnets (*p*_C_/*p*_S_ > 1). A comparison between Co_2_VGa and Co_2_MnGa indicates that the alloy with a larger spin polarization showed a smaller *p*_C_/*p*_S_ value. Further, an experimental investigation into the temperature dependence of spin polarization is needed to clarify the mechanism of shrinkage of the magnetic moments in the paramagnetic phase at a high temperature.

## Figures and Tables

**Table 1:** The magnetic parameters of Ni_2+*x*_MnGa_1−*x*_ (*x* = 0.00, 0.02, 0.04). The spontaneous magnetic moment, *p*_S_; effective moment, *p*_eff_; Curie temperature, *T*_C_; spin fluctuation parameter in *k*-space (momentum space) *T*_A_, and that in energy space, *T*_0_. The parameters *T*_A_ (*T*_C_) and *T*_0_ (*T*_C_) were obtained from the *M*^4^ vs. *H/M* plot at *T*_C_ [9]. The *p*_eff_, *T*_A_ (5 K) and *T*_0_ (5 K) were the obtained values in this work.

Sample	*p*_S_ (*μ*_B_/f. u.)	*p*_eff_ (*μ*_B_/f. u.)	*T*_C_ (K)	*T*_A_ (K) (5 K)	*T*_A_ (K) (*T*_C_) [9]	*T*_0_ (K) (5 K)	*T*_0_ (K) (*T*_C_) [9]
Ni_2_MnGa	3.93	4.75	375	556	563	254	245
Ni_2.02_MnGa_0.98_	3.79	4.72	372	580	566	269	288
Ni_2.04_MnGa_0.96_	3.64	4.68	366	583	567	316	345

**Table 2:** Basic magnetic parameters and *k*_m_ as obtained from Equation (8).

	*T*_C_ (K)	*p*_eff_ (*μ*_B_)	*p*_S_ (*μ*_B_)	*p*_eff_/*p*_S_	*T*_A_ (K)	*T*_0_ (K)	*T*_C_/*T*_0_	*k*_m_	Reference ^1^
Ni_2_MnGa	375	4.75 *	3.93	1.21	563	245	1.53	1.61	This work *, [9]
Ni_2.02_MnGa_0.98_	372	4.72 *	3.79	1.25	566	288	1.29	1.48	This work *, [9]
Ni_2.04_MnGa_0.96_	366	4.68 *	3.64	1.28	567	345	1.06	1.34	This work *, [9]
Co_2_VGa	337	2.06	1.87	1.10	2258	213	1.58	1.50	This work
Co_2_MnGa	695	4.16	4.09	1.02	1,037	364	1.91	1.57	[16,19]
Ni	623	3.3	0.6	5.5	1.76 × 10^4^	4.83 × 10^3^	0.129	1.41	[13]
MnSi	30	2.25	0.4	5.6	2.18 × 10^3^	155	0.194	1.88	[7,10]
Ni_3_Al	41.5	1.3	0.075	17.3	3.67 × 10^4^	2.76 × 10^3^	0.015	1.06	[7,20]
Y(Co_0.85_Al_0.15_)_2_	26	2.15	0.138	15.6	7.26× 10^3^	1.41× 10^3^	0.018	1.08	[7,21]
ZrZn_2_	21.3	1.44	0.12	12	7.4× 10^3^	1390	0.015	0.74	[7,22]
CoS_2_	120	1.72	0.98	1.76	2.20× 10^3^	294	0.41	0.96	[12,23]
UCoGe	2.4	1.93	0.039	49.5	5.92 × 10^3^	362	0.0065	1.74	[7,24]
UGe_2_	52.6	3.00	1.41	2.13	442	92.2	0.571	1.61	[14]
NpFe_4_P_12_	23	1.55	1.35	1.15	285	16.4	1.40	1.44	[14,25]

^1^ Citations in our published paper [9] are incorrect. The correct citations are listed above. We apologize for this mistake. * These values were obtained by this work.

**Table 3:** Magnetic parameters of ferromagnetic Heusler alloys. *p*_C_ indicates the magnetic moment at the paramagnetic phase. The relation between *p*_eff_ and *p*_C_ is defined by the equation of $p_{\mathrm{eff}}=\sqrt{p_{C}\left(p_{C}+2\right)}$.

Sample	*T*_C_ (K)	*p*_S_ (μ_B_/f.u.)	*p*_eff_ (μ_B_/f.u.)	*p*_C_ (μ_B_/f.u.)	*p*_C_/*p*_S_	Reference
Ni_2_MnGa	375	3.93	4.75 *	3.85	0.980	This work *, [9]
Ni_2.02_MnGa_0.98_	372	3.79	4.72 *	3.82	1.01	This work *, [9]
Ni_2.04_MnGa_0.96_	366	3.64	4.68 *	3.79	1.04	This work *, [9]
Co_2_VGa	337	1.87	2.06	1.30	0.70	This work
Co_2_MnSi	1034	5.01	2.86	2.03	0.41	[30]
Co_2_MnGe	905	4.76	3.70	2.82	0.56	[30]
Co_2_MnSn	825	5.02	5.29	4.38	0.87	[31]
Co_2_MnGa	695	4.09	4.16	3.28	0.80	[19]
Co_2_FeSi	1015	5.42 (300 K)	5.65	4.74	0.875	[32]
Co_2_FeGa	1089	5.05 (300 K)	4.59	3.69	0.730	[32]
CoMnSb	478	4.2	4.0–4.6	3.1-3.7	0.74–0.88	[27]
NiMnSb	728	4.2	2.9–4.2	2.1-3.3	0.69–0.79	[27]
PtMnSb	572	3.96	4.3–4.9	3.4-4.0	0.86–1.01	[27]
Ni_2_MnIn	315	4.4	4.69	3.80	0.86	[33,34]
Rh_2_MnSn	410	4.14	4.83	3.93	0.95	[31]
Pd_2_MnSn	189	4.23	4.70	3.81	0.90	[28]
Pd_2_MnSb	255	4.40	4.8	3.9	0.89	[28]

* These values were obtained by this work.
